# Efficacy of Exercise on Postneedling Soreness: A Randomized Controlled Trial

**DOI:** 10.3390/jcm10235527

**Published:** 2021-11-25

**Authors:** Nicola Sante Diciolla, Celia Pérez-Clemente, Marta Cámara-Caballero, Alberto Matienzo-Barreto, Alba Real-Rodríguez, María Torres-Lacomba

**Affiliations:** 1 Physiotherapy in Women’s Health (FPSM) Research Group, Physiotherapy Department, Faculty of Medicine and Health Sciences, University of Alcalá, 28805 Madrid, Spain; maria.torres@uah.es; 2 Independent Researcher, 19001-5 Guadalajara, Spain; perez.clemente.celia@gmail.com; 3 Independent Researcher, 28001-20 Madrid, Spain; martacamaracaballero@gmail.com (M.C.-C.); alber.matienzo@gmail.com (A.M.-B.); 4 Faculty of Physiotherapy, University of A Coruña, 15006 A Coruña, Spain; albarealrodriguez@gmail.com

**Keywords:** pain, trigger points, physical therapy, dry needling, therapeutic exercise

## Abstract

This study aimed to investigate the efficacy of concentric, eccentric, and isometric exercise protocols on the postneedling soreness (PNS) after the dry needling (DN) of latent myofascial trigger points (MTrP) in the medial gastrocnemius muscle. A randomized clinical trial was carried out. Volunteers, ≥18 years old, with a latent MTrP in the medial gastrocnemius muscle were included. Subjects with contraindications to DN, active MTrPs, and/or other treatments in MTrPs in the 3 months prior to recruitment were excluded. A total of 69 participants were randomly allocated to four groups, where post-DN intervention consisted of an eccentric, concentric, or isometric exercise, or no exercise, and they were assessed for PNS intensity (visual analog scale (pVAS)), pressure pain threshold (PPT, analog algometer), pain intensity (nVAS), and local twitch responses (LTRs) during DN, as well as demographics and anthropometrics. The mixed-model analyses of variance showed significant interaction between time and pVAS, and between time and PPT (*p* < 0.001). While the multivariate test confirmed that PNS and PPT improved over time within each group, specifically between 6–12 h post-intervention, the *post hoc* analyses did not show significant differences between groups. The mixed-model analyses of covariance showed a significant nVAS effect (*p* < 0.01) on PNS decrease, and some effect of the LTRs (*p* < 0.01) and sex (*p* = 0.08) on PPT changes. All groups improved PNS and PPT, but none of them showed a greater improvement above the others. The most dramatic decrease was observed between 6–12 h post-exercise, although concentric and eccentric exercise had an effect immediately after the intervention. Between all potential modifiers, pain during DN significantly influenced PNS progression, while LTRs and sex seemed to determine PPT course over time.

## 1. Introduction

The myofascial trigger point (MTrP) defines an irritable area within a skeletal muscle associated with a hyperalgesic nodule located in a tight band of muscle fibers, and can be prone to painful mechanical deformations (i.e., contraction, stretching, compression) [1,2]. Depending on its clinical form, MTrP may cause referred symptomatic patterns of pain, motor dysfunction, and autonomic phenomena. However, active MTrP can cause major disorders in combination with spontaneous referred pain [1].

Among the different therapeutic approaches, dry needling (DN) appears to be the most appropriate option for the treatment of MTrPs, as it improves pain (i.e., by decreasing intensity and increasing the pressure pain threshold), range of motion, and quality of life in the short- and medium-term, compared with placebo and/or no intervention [3,4,5].

DN consists of inserting a needle through the skin, without injecting or extracting any substance, in order to trigger a mechanical stimulus and the associated responses from the peripheral and central nervous systems [6]. At the same time, this action can produce mild to moderate side effects, the most notable of which is postneedling soreness (PNS). This results from damage to neuromuscular tissue, caused by the needle, which triggers an immediate inflammatory response [7]. All subjects undergoing DN report PNS [8], with an average duration of less than 72 h [9].

Factors and determinants for PNS have already been studied. While a higher amount of needle insertions during DN and female gender in latent MTrPs [10], as well as a higher dose of local twitch responses (LTRs ≥ 6) in active MTrPs [11] seem to increase the intensity, psychological factors such as catastrophism may decrease the intensity, but delay resolution [12]. As a result, PNS may limit the activities of daily living, overlie the symptoms of the individual, and reduce therapeutic adherence [13].

To prevent this scenario, several strategies have been proposed for the management of PNS [14,15,16,17]. Percutaneous electrical nerve stimulation reduces PNS intensity, if applied after DN of active MTrPs in the upper trapezius muscle in patients with chronic neck pain [14]. Alternatively, cold spray with stretching seems to reduce PNS symptoms in the short-term (<6 h) [15], while ischemic compression lowers both its intensity and duration [16] after DN of latent MTrPs in the upper trapezius muscle.

Recently, after DN of active MTrPs in the infraspinatus muscle of subjects with subacromial pain syndrome, low-load eccentric exercise was performed, which reduced pain intensity immediately after the DN, at 24 h and 48 h, compared with the placebo or no intervention [17]. To the authors knowledge, however, this is the only study dealing with the management of PNS by exercise, and the current literature seems to focus on tendinopathies (which could be somehow related to MTrP). Nevertheless, no conclusive recommendations regarding the most effective exercise modality were found [18,19,20,21,22].

The eccentric mode does not seem to be superior to the concentric mode, neither for reducing pain, nor for increasing functionality in Achilles tendinopathy, although it is included in various protocols [18,19]. Simultaneously, the isometric mode would be the best choice in cases with patellar tendinopathy, and not the eccentric mode [20]. Eccentric exercise could relieve pain and increase strength in cases of tendinosis of the lower and upper limbs, but its superiority over other exercise modes is questionable [21,22].

To resume exercise in its different modalities seems to be beneficial in the management of acute pain; however, the lack of studies showing these effects on PNS justified a randomized clinical trial, which aimed to determine the efficacy of concentric, eccentric, and isometric exercise protocols on the PNS after DN of latent MTrPs in the medial gastrocnemius muscle. Furthermore, we wanted to study the outcomes which could, a priori, influence PNS progression (pain during DN, amount of LTRs, and sex).

## 2. Materials and Methods

### 2.1. Study Design

A randomized single-blind clinical trial with parallel groups to compare the effects of eccentric, concentric, or isometric exercises (three experimental groups) with no intervention (control group) on PNS. The study was reported according to the Consolidated Standards of Reporting Trials (CONSORT) statement [23]. This project was approved by the Ethics Committee on Animal and Human Experimentation of the University of Alcalá (CEI/HU/2019/27) and registered with clinicaltrial.gov (NCT04135339). All subjects gave their informed consent; anonymity and confidentiality were guaranteed through the encoding of files, following the current regulation and by complying with the Declaration of Helsinki.

### 2.2. Participants

Volunteers, ≥18 years old, without obvious symptoms in the triceps surae muscle, were recruited in different physiotherapy clinics with home-visit services in Madrid, Spain.

Subjects with at least one latent MTrP in the medial gastrocnemius muscle (MTrP1) were included [24]. Participants were diagnosed with latent MTrP if there were: (a) a palpable tight band in the muscle, (b) a hypersensitive and hyperalgesic nodule in the tight band, and (c) painful limitations to full stretch. Both (a) and (b) were the most reliable essential diagnostic criteria for identifying MTrPs [2,25]. A significant correlation between latent MTrPs, the prevalence of tight bands and tender points in the gastrocnemius muscles had been reported [26]. Those subjects with pathology in the legs that prevented the application of the proposed exercise protocols (e.g., fractures in the legs, sprain in the acute phase), conditions associated with active MTrPs in the medial gastrocnemius muscle (e.g., Achilles tendinopathy, plantar fasciitis), fibromyalgia, coagulation disorders, osteosynthesis material, pregnancy status, fear of needles (i.e., belonephobia), previous application of DN, or any intervention in MTrPs of gastrocnemius muscle in the 3 months pre-recruitment were excluded.

### 2.3. Dry Needling Protocol

All subjects received DN in a latent MTrP in the medial gastrocnemius muscle (MTrP1) in prone position, according to the protocol proposed by Mayoral and co-workers [24]. The same physiotherapist (Pt1 or Pt2) diagnosed and applied DN. When more than one latent MTrP was found, the most painful was selected. Once the MTrP was located, it was marked with an indelible dermographic marker, while the skin was cleaned and disinfected with an alcoholic solution of 2% chlorhexidine. Before inserting the needle, subjects were warned about the possible pain experience and/or LTR during the intervention. DN was performed with a 0.30 × 50 mm solid filament needle [Agupunt, Agujas Punción Seca (APS^®^), Barcelona, Spain] inserted through the skin into the MTrP in the taut band, resulting in a first LTR. Next, the needle was moved in and out of the MTrP using Hong’s Fast-In and Fast-Out technique [8], up to 50 insertions [12]. When the needle was withdrawn, the area was compressed firmly for approximately 90 s, applying a pressure of 4 kg/cm^2^. The amount of LTRs was recorded, since a higher dose could lead to more severe PNS [11].

### 2.4. Post-Needling Intervention

After DN, participants were randomly allocated to four groups: three experimental groups, which immediately performed a single eccentric, concentric, or isometric exercise protocol, and a control group, which had no further intervention. Pt1 or Pt2 followed the exercise session.

The exercise protocol was based on the proposal by Alfredson et al. [27]. The same working scheme was followed in the three experimental groups. The subjects were instructed to perform 3 sets of 15 repetitions (3 s each) with 30 s breaks between the sets with the leg to which DN was applied. The participants reached the starting position with the other leg in order to obtain the purest contractions.

### 2.5. Assessment Procedure

#### 2.5.1. Post Needling Soreness (PNS)

It was quantified using a visual analog scale (pVAS) of 100 mm, consisting of a 100 mm horizontal line with pain descriptors marked “no pain” on the left side and “the worst imaginable pain” on the right side [28]. The assessments were performed 2 min after DN, after the exercise protocol, at 6 h, 12 h, 24 h, 48 h, and 72 h after the intervention. VAS has already shown a high reliability for acute pain (ICC = 0.97; 95% CI 0.96–0.98) [29].

#### 2.5.2. Pressure Pain Threshold (PPT)

The PPT was evaluated with an analogue pressure algometer (Wagner Instruments, Greenwich, CT, USA) before DN, 2 min after DN, after the exercise session, at 24 h, and at 48 h after the intervention. PPT was defined as the minimum amount of pressure required to cause pain. Three consecutive tests of PPT were performed on the MTrP at a speed of 1 kg/s with intervals of 30 s; the average value was computed using at least two measurements, with a variability of less than 5–10%. The pressure algometry provided a valid and reliable measurement for PPT [30].

#### 2.5.3. Outcomes Which Potentially Influence PNS

Data were recorded regarding pain (nVAS) and the amount of LTRs during DN.

#### 2.5.4. Medication Intake

Each event and type of medication use was recorded, although participants were asked to avoid the use of drugs during the three days of follow-up.

#### 2.5.5. Demographics (Sex, Age) and Anthropometrics

Demographics (sex, age) and anthropometrics (weight, height) were collected.

### 2.6. Study Protocol

Firstly, DN was applied to the latent MTrP that had been previously diagnosed. Next, the subjects were randomly allocated into four different groups using a randomization program: three experimental groups, which performed a concentric, eccentric, or isometric exercise protocol, and a control group without further intervention.

Two VASs were used: one related to pain during DN (nVAS), and another for PNS (pVAS). Afterwards, pVAS was recorded 2 min after DN, after the exercise session, and again at 6 h, 12 h, 24 h, 48 h, and 72 h after the intervention. Alternatively, the PPT score was recorded before and 2 min after DN, after the exercise session, at 24 h, and 48 h after the intervention. All outcomes were registered by the same physiotherapist (Pt3) who dealt exclusively with the assessments and was blinded to the group allocation of the participants.

### 2.7. Sample Size Calculation

The computation of the sample size was carried out with the software G*Power 3.1.9.4. The pVAS was chosen as the primary outcome, and its effect was estimated to be wide (0.5). Considering a power of 0.95, a α error of 0.05, a correlation between repeated measures of 0.5, and a non-sphericity correction of 1.0, at least 12 subjects/group were required (number of groups = 4; number of assessments = 7; ANOVA statistical test: repeated measures, within-between interaction). Allowing for 20% of dropouts/losses, at least 15 subjects/group were required.

### 2.8. Randomization

The participants were randomly allocated to the different groups by another researcher (Pt4) who was not involved in the fieldwork (data collection or intervention) using the block randomization method (GraphPad Software, Inc., La Jolla, CA, USA).

### 2.9. Masking

The outcomes assessor (Pt3) was kept blinded after the randomization.

### 2.10. Statistical Analyses

Statistical analyses were performed using IBM SPSS Statistics for Windows, v.22 (IBM Corp., Armonk, NY, USA).

The data were expressed as mean and standard deviation (mean(SD)) or median and interquartile range (median(Q1–Q3)), depending on the distribution obtained by the Shapiro-Wilk test and the visual inspection of the histograms. For the categorical variables (gender), absolute and relative frequencies were used. One-way analysis of variance (ANOVA) for continuous outcomes and a χ^2^ test for categorical data were performed to examine differences between groups at baseline.

#### 2.10.1. To Investigate the Effects of Each Intervention on PNS and PPT

For PNS, a 7 × 4 mixed ANOVA was performed with time (2 min after DN, after the exercise session, at 6 h, 12 h, 24 h, 48 h, and 72 h) as within-subjects factor, and group (eccentric, concentric, and isometric exercise group, or control group) as between-subjects factor.

For PPT a 5 × 4 mixed ANOVA was run with time (before and 2 min after DN, after the exercise session, at 24 h, and 48 h) as within-subjects factor and the group as between-subjects factor. Post hoc analyses were conducted using Bonferroni corrections (*p* < 0.0125).

#### 2.10.2. To Assess Whether nVAS, the Amount of LTRs, and Sex Have Some Influence on Changes in PNS within Each Group

Mixed analyses of covariance (ANCOVA) were proposed, including nVAS, the amount of LTRs, and sex as covariates.

We performed a 7 × 4 mixed ANCOVA for PNS and a 5 × 4 ANCOVA for PPT, maintaining the same within-subjects and between-subjects factors we had previously used for mixed-model ANOVA.

## 3. Results

**Figure 1:**
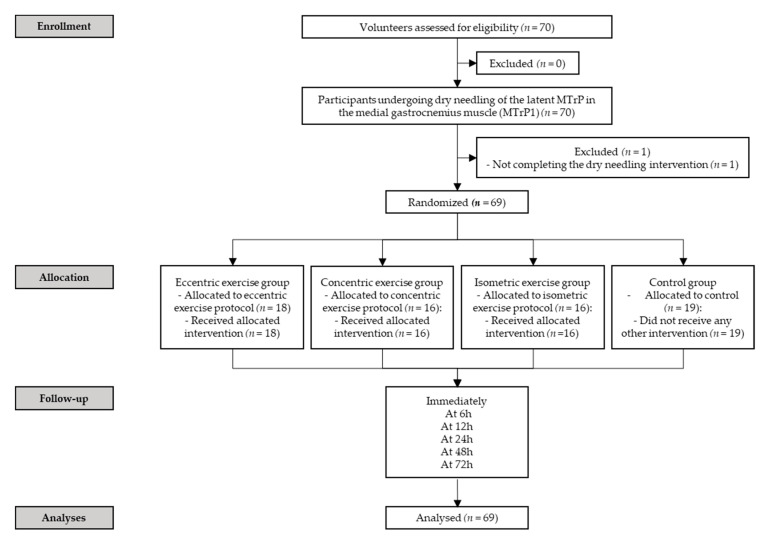
Flowchart of participants throughout the trial; MTrP (myofascial trigger point).

Seventy (*n* = 70) subjects were screened for eligibility criteria. Sixty-nine satisfied the eligibility criteria, agreed to participate, and were randomized into eccentric exercise (*n* = 18), concentric exercise (*n* = 16), isometric exercise (*n* = 16) and control (*n* = 19) group. One subject did not meet the inclusion criteria and, therefore, was excluded (Figure 1). No adverse effects were found in either the three intervention or the control groups.

No statistically significant differences were found between groups at baseline on almost all variables collected (Table 1).

### 3.1. Post-Needling Soreness and Pressure Pain Threshold

The 7 × 4 mixed-model ANOVA showed a significant interaction between time and pVAS *F*(3,199) = 61.33, *p* < 0.001, *η*^2^ = 0.48. On the other hand, no significant time*group interaction was found (*p* = 0.26), and tests of effect between subjects confirmed no significant main effect of group on (*p* = 0.48). Therefore, by ignoring other outcomes there was no statistical differences in pVAS according to the group.

Pairwise comparisons per time showed significant statistical differences in pVAS between different time points (*p* < 0.001), specifically between 6–12 h, 12–24 h, 24–48 h, and 48–72 h. As such, starting from 6 h–12 h after intervention, PNS decreased drastically. Multivariate test confirmed PNS decreased over time; *Wilks’ λ* = 0.30, *F*(6,60) = 23.28, *p* < 0.001, and *η*^2^ = 0.70.

Multiple pairwise comparisons per time*group, adjusting for group, showed no significant differences in pVAS within each time point. However, just at 72 h, there was a statistically significant difference between the isometric and eccentric group (*p* = 0.03), and a trend to statistically significant *p* between the isometric and concentric group (*p* = 0.08), as well as between the isometric and control group (*p* = 0.06).

Multiple pairwise comparisons per time*group, adjusting for time, showed many statistically significant differences in pVAS within each group. Specifically, between consecutive assessments, pVAS showed a dramatic decrease between 6–12 h after intervention (*p* = 0.02) in the eccentric group, and between 6–12 h (*p* = 0.03), 12–24 h (*p* < 0.001), and 24–48 h (*p* < 0.001) in the control group. However, when comparing the first assessment (2 min after the DN) to the others, PNS started decreasing significantly at 24 h in the eccentric group (*p* < 0.01), at 12 h in the concentric group (*p* = 0.04), at 24 h in the isometric group (*p* = 0.03), and at 12 h in the control group (*p* = 0.01).

**Figure 2:**
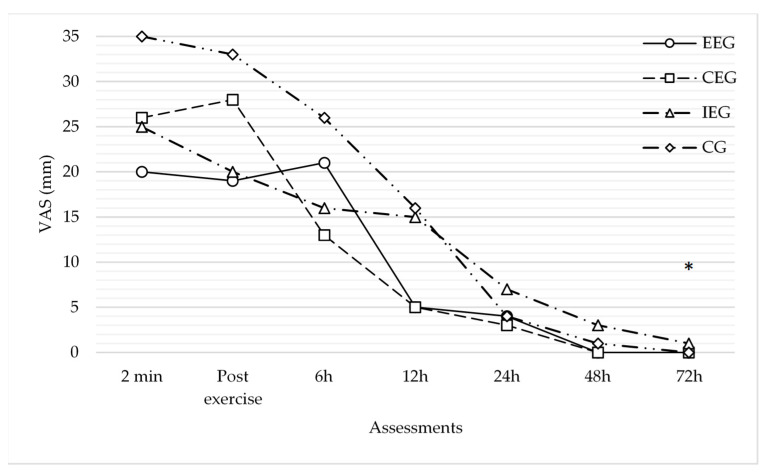
Comparison analysis of the change in the postneedling soreness intensity over time between groups. * Statistically significant *p*. *p*-values proceeding from multiple pairwise comparisons per time*group, adjusting for group (mixed ANOVA); comparisons between groups in each assessment (based on estimated marginal means). EEG, eccentric exercise group; CEG, concentric exercise group; IEG, isometric exercise group; CG, control group; VAS, visual analogue scale.

Multivariate test confirmed PNS decreased over time within each group (eccentric group: *Wilks’ λ* = 0.64, *F*(6,60) = 5.65, *p* < 0.01, and *η*^2^ = 0.36; concentric group: *Wilks’ λ* = 0.61, *F*(6,60) = 6.22, *p* < 0.001, and *η*^2^ = 0.38; isometric group: *Wilks’ λ* = 0.73, *F*(6,60) = 3.61, *p* < 0.001, and *η*^2^ = 0.26; control group: *Wilks’ λ* = 0.46, *F*(6,60) = 11.60, *p* < 0.001, and *η*^2^ = 0.54). However, *post hoc* analyses showed no significant differences between the groups in their PNS decrease. Therefore, no intervention group exhibited a higher decrease in PNS above the others (Table 2; Figure 2).

The 5 × 4 mixed-model ANOVA showed a significant interaction between time and PPT *F*(3,170) = 6.53, *p* < 0.001, and *η*^2^ = 0.09. Furthermore, a significant time*group interaction was found (*F*(8) = 2.11, *p* = 0.04, and *η*^2^ = 0.08). However, tests of effects between-subjects did not confirm a significant main effect of group on PPT (*p* = 0.35). Therefore, by ignoring other outcomes, there was no statistical differences in PPT according to the group.

Multiple pairwise comparisons per time*group, adjusting for group, showed no significant statistical differences in PTT within each time point.

Alternatively, multiple pairwise comparisons per time*group, adjusting for time, showed many statistically significant differences in PPT within each group. Specifically, between consecutive assessments, PPT showed a significant change between 24–48 h after intervention (*p* < 0.01) in the eccentric group, and between 24–48 h (*p* < 0.01) in the concentric group. However, when comparing the first assessment (before the DN) to the others, no significant change was found (*p* > 0.05).

**Figure 3:**
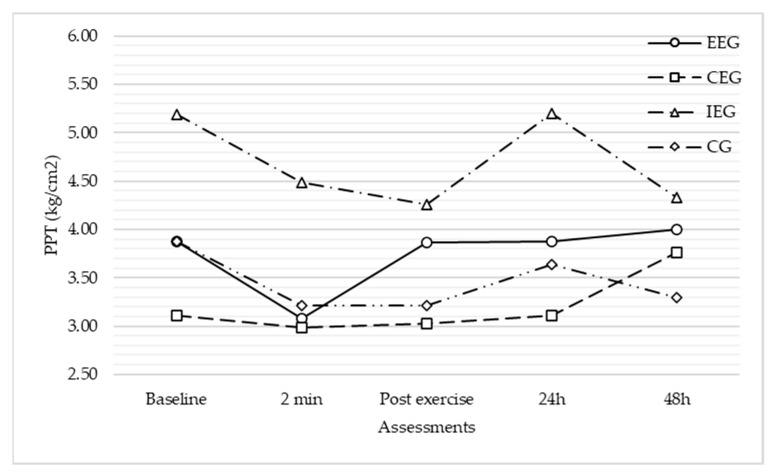
Comparison analysis of the change in the pressure pain threshold over time between groups. No differences between groups within each assessment (*p* > 0.05). *p*-values proceeding from multiple pairwise comparisons per time*group, adjusting for group (mixed ANOVA); comparisons between groups in each assessment (based on estimated marginal means). EEG, eccentric exercise group; CEG, concentric exercise group; IEG, isometric exercise group; CG, control group; PPT, pressure pain threshold.

Multivariate test showed significant changes in PPT over time within each group (eccentric group: *Wilks’ λ* = 0.75, *F*(4,62) = 5.23, *p* < 0.01, and *η*^2^ = 0.25; concentric group: *Wilks’ λ* = 0.78, *F*(4,62) = 4.25, *p* < 0.01, and *η*^2^ = 0.21; and the isometric group: *Wilks’ λ* = 0.75, *F*(4,62) = 5.03, *p* < 0.01, and *η*^2^ = 0.24), apart from the control group: *Wilks’ λ* = 0.89, *F*(4,62) = 1.97, *p* = 0.11, and *η*^2^ = 0.11. However, *post hoc* analyses showed no significant differences between groups. Therefore, no intervention group exhibited a higher change in PPT above the others (Table 3; Figure 3).

### 3.2. Effects of Pain during the DN, Amount of LTRs, and Sex

The 7 × 4 mixed-model ANCOVA showed a significant time*nVAS interaction *F*(3,192) = 9.26, *p* < 0.001, and *η*^2^ = 0.14, with no effect of the amount of LTRs (*p* = 0.29), the group (*p* = 0.80), the sex (*p* = 0.30), or the group*sex interaction (*p* = 0.86), for changes in PNS. Furthermore, tests of effects between subjects confirmed the effect of nVAS (*p* < 0.01), and no effects of group, sex, or group*sex interaction (*p* > 0.05), but instead highlighted the effect of the amount of LTRs (*p* = 0.04) for changes in PNS.

Multivariate test confirmed PNS decreased over time within each group (eccentric group: *Wilks’ λ* = 0.55, *F*(6,54) = 7.37, *p* < 0.001, and *η*^2^ = 0.45; concentric group: *Wilks’ λ* = 0.51, *F*(6,54) = 8.65, *p* < 0.001, and *η*^2^ = 0.49; isometric group: *Wilks’ λ* = 0.57, *F*(6,54) = 6.84, *p* < 0.001, and *η*^2^ = 0.43; and control group: *Wilks’ λ* = 0.46, *F*(6,54) = 10.70, *p* < 0.001, and *η*^2^ = 0.54), and decreased over time within each sex (female: *Wilks’ λ* = 0.35, *F*(6,54) = 16.54, *p* < 0.001, and *η*^2^ = 0.65; male: *Wilks’ λ* = 0.36, *F*(6,54) = 16.09, *p* < 0.001, and *η*^2^ = 0.64) (pairwise comparisons including covariates -LTRs and nVAS-).

The 5 × 4 mixed-model ANCOVA still showed a trend to statistically significant time*group interaction (*p* = 0.07) and time*sex interaction (*p* = 0.08), with no effect of nVAS (*p* = 0.835), the amount of LTRs (*p* = 0.61), or group*sex interaction (*p* = 0.77) for changes in PPT. Simultaneously, tests of effects between subjects confirmed some effect of the sex (*p* = 0.07) and no effects of nVAS, group, or group*sex interaction (*p* > 0.05), but highlighted the effect of the amount of LTRs (*p* < 0.001) for changes in PPT.

Multivariate test confirmed changes in PPT over time within each group (eccentric group: *Wilks’ λ* = 0.72, *F*(4,56) = 5.33, *p* < 0.001, and *η*^2^ = 0.28; concentric group: *Wilks’ λ* = 0.78, *F*(4,56) = 4.05, *p* < 0.001, and *η*^2^ = 0.22; and the isometric group: *Wilks’ λ* = 0.78, *F*(4,56) = 3.84, *p* < 0.001, and *η*^2^ = 0.21) apart from the control group: *Wilks’ λ* = 0.91, *F*(4,56) = 1.42, *p* = 0.24, and *η*^2^ = 0.09) and changed over time within each sex (female: *Wilks’ λ* = 0.63, *F*(4,56) = 8.24, *p* < 0.001, and *η*^2^ = 0.37; male: *Wilks’ λ* = 0.78, *F*(4,56) = 3.98, *p* < 0.001, and *η*^2^ = 0.22) (pairwise comparisons including covariates -LTRs and nVAS-).

## 4. Discussion

Our study aimed to investigate the effects of different exercise modalities on PNS and PPT of latent MTrPs, apart from studying pain and the amount of LTRs during DN, and sex as potential modifiers of their course.

All groups experienced a decrease in PNS over time, which was dramatically pronounced between 6–12 h after the intervention. Alternatively, the no intervention group showed a greater decline in PNS above the others. However, as a qualitative estimate, the concentric exercise group seemed to have an earlier effect on PNS immediately after the intervention. Considering all potential modifiers, pain during DN significantly influenced PNS progression over time, above the amount of LTRs and sex. However, the LTRs appeared to have an effect, while sex had none.

At the same time, PPT had a similar progression over time in the different groups and the no intervention group exhibited a higher change in PPT above the others. However, a group effect was found, in which the restoration of PPT to the baseline level could be explained immediately after the exercise session in the eccentric and concentric exercise groups. By considering all potential modifiers, LTRs and sex seemed to drive an effect on PPT progression over time, while pain during DN showed none.

### 4.1. Comparison with Previous Studies

Our analysis did not allow us to find significant differences in the progression of PNS or PPT after the application of the different contraction modalities.

On a qualitative level, however, a dramatic decrease in PNS intensity was observed as early as 6 h after the intervention in the concentric exercise group, and 12 h after the intervention in the eccentric exercise group, exceeding the cut-off of 10 mm, indicating a real change in the pain experience [29]. In accordance with our results, Salom-Moreno et al. showed that a low-load exercise protocol, combining concentric and eccentric contractions, caused a huge decrease in PNS intensity, exceeding the minimal clinically important difference in pain intensity after the intervention [17]. However, their sample included only subjects with active MTrPs. Interestingly, the PNS reduction observed after concentric and eccentric exercises in our study was similar to the previously reported reduction when using spray and stretch or ischemic compression in latent MTrPs [15,16].

The other groups achieved this result only 24 h after the intervention.

Salom-Moreno et al. transferred their results to a greater efficacy of low-load exercise compared to a placebo (i.e., disconnected ultrasound) and no intervention in PNS progression. However, their study did not consider other exercise modalities, because they assumed that their exercise proposal was more effective than others [17].

Alternatively, in previous studies investigating the efficacy of different exercise modalities in clinical contexts with pain, other than myofascial syndromes (e.g., tendinopathies), no conclusive recommendations were found, because none of the exercise modalities proved more effective than the others [18,19,20,21,22], which was consistent with our results. Potentially, the choice of exercise mode should be determined by other individual factors, such as pain location, duration, patient preference, or tolerance.

Regarding potential modifiers of PNS, we found that pain during DN and the number of LTRs influenced PNS progression, which was consistent with Martín-Pintado et al., who showed that pain during DN correlated significantly with PNS, and that higher doses in LTRs during DN correlated with higher VAS scores for PNS 24 h and 48 h after intervention, when compared with lower doses [10,11].

In contrast, sex did not show much influence on PNS, which was still discussed in the literature [10,17]. Nevertheless, when the groups were sorted by sex and based on our data (we estimated the marginal means adjusting for nVAS and the amount of LTRs), PNS course seemed very similar, except for the eccentric exercise group in males.

Finally, regardless of the intervention used, all groups experienced a significant improvement in pVAS, which decreased between 6–12 h after the intervention and completely at 48–72 h after the intervention, in line with previous studies [10,15,16,17].

Regarding the progression of PPT, each group presented a different scenario. The eccentric and concentric exercise group showed a certain decrease after DN (as expected) and an increase to the pre-needling level immediately after the exercise intervention. In the concentric group, PPT increased above the starting level 48 h after the intervention. In the isometric exercise and control groups, however, PPT did not reach the baseline level until 24 h after the intervention. These results contrast with previous studies, in which PPT did not reach the initial level until 48 h after DN alone or in combination with spray and stretch [10,15]. Therefore, we could hypothesize that different exercise proposals could restore PPT earlier when compared with other interventions or no intervention after DN. Further research is required regarding this issue. Nevertheless, although time*group interaction was observed, this effect was not confirmed.

When considering potential modifiers of PPT, we found an effect of sex and amount of LTRs. However, this effect was not confirmed and, sorting groups by sex (adjusting for nVAS and the amount of LTRs), we found similar patterns over PPT course. In line with our results, Martín-Pintado et al. already suggested no effect of sex for changes in PPT [10].

### 4.2. Clinical Importance

Finding the best strategy for the management of PNS would make the experience of DN less uncomfortable, more tolerable and could reduce and/or avoid the rejection of this intervention [13], which would broaden therapeutic options for pain of myofascial origin, since short and medium-term clinical effectiveness of DN is known [3,4,5].

All types of training seem to improve the PNS and PPT between 6–12 h after the intervention, when performed at the same intensity as suggested in our protocol. Eventually, concentric exercise may influence the PNS immediately after the intervention, while eccentric exercise may return PPT to baseline level immediately after the exercise session. However, pain during DN, LTRs, and sex could alter the course of both outcomes over time. Thus, the choice of training modality should be determined by other factors and this study could also help in this issue.

### 4.3. Strengths and Limitations

To the best of our knowledge, this is the first study attempting to analyse the efficacy of different forms of exercise, differentiated by dominant muscle contraction (eccentric, concentric, and isometric), in order to modify PNS progression.

However, some limitations must be highlighted.

Firstly, it was not possible to mask the participants in the post-DN intervention.

Secondly, our study counted only subjects with latent MTrPs; therefore, the present results cannot be applied to subjects with active MTrPs who might be more symptomatic. Furthermore, DN was applied to only one MTrP of the selected muscle, and we ignored whether the same results would be obtained if the same intervention was applied to several MTrPs and/or different muscles simultaneously (which is quite common in clinical practice).

In contrast, it was not possible to prevent the participants from performing other exercise modalities, although they were advised to avoid activities, such as walking or running, during the follow-up in order not to influence the results of the interventions.

Finally, psychological outcomes, which are already known to affect PNS progression [12], were not recorded. However, these were not related to the purpose of our study.

Future research should be conducted with these limitations in mind.

## 5. Conclusions

All interventions improved PNS and PPT, but none of them showed a greater improvement above the others. The most dramatic decrease in PNS was observed between 6–12 h after the intervention, although concentric exercise had an effect immediately after the intervention. Eccentric exercise brought PPT back to its baseline level immediately after the exercise session. Considering all potential modifiers, pain during DN significantly influenced PNS progression, while LTRs and sex seemed to determine the course of PPT over time.

## Figures and Tables

**Table 1:** Baseline characteristics of participants.

	Eccentric Exercise Group (*n* = 18)	Concentric Exercise Group (*n* = 16)	Isometric Exercise Group (*n* = 16)	Control Group (*n* = 19)	Sig.
Demographics					
Sex (male) *n* (%)	10 (56)	9 (56)	6 (37)	10 (53)	*p* = 0.68
Age (years)	34 (13)	34 (13)	34 (14)	36 (14)	*p* = 0.94
Anthropometrics					
Weight (kg)	73 (15)	71 (13)	69 (14)	74 (14)	*p* = 0.67
Height (m)	1.73 (0.09)	1.72 (0.08)	1.70 (0.09)	1.73 (0.10)	*p* = 0.75
Clinical Assessments					
nVAS (mm)	45 (24)	47 (16–61)	32 (23)	54 (21)	*p* = 0.04 *
PPT before DN (kg/cm^2^)	3.87 (1.34)	3.11 (2.86–5.05)	5.19 (2.38)	3.87 (1.37)	*p* = 0.12
LTRs (*n*)	12 (8)	6 (5–20)	6 (2–15)	6 (4–15)	*p* = 0.86

Data were expressed as mean (SD) or median (Q1–Q3), unless otherwise stated. One-way ANOVA for continuous outcomes and *χ*^2^ test for categorical data were performed. * Statistically significant *p*. nVAS, visual analogue scale for pain during dry needling; DN, dry needling; PPT, pressure pain threshold; LTR, local twitch response.

**Table 2:** Change in postneedling soreness intensity (pVAS, mm) over time within each group.

	2 min after	Post Exercise	6 h after	12 h after	24 h after	48 h after	72 h after	Sig.
Eccentric Exercise Group	20 (30–10)	19 (8–23)	21 (9–34)	5 (3–21)	4 (1–14)	0 (0–0)	0 (0–0)	*p* < 0.001 *
Concentric Exercise Group	26 (20)	28 (21)	13 (4–43)	5 (3–14)	3 (0–4)	0 (0–4)	0 (0–1)	*p* < 0.001 *
Isometric Exercise Group	25 (19)	20 (6–48)	16 (6–40)	15 (6–27)	7 (3–11)	3 (0–8)	1 (0–5)	*p* < 0.01 *
Control Group	35 (19)	33 (20)	26 (6–63)	16 (4–39)	4 (0–17)	1 (0–4)	0 (0–1)	*p* < 0.001 *

Data were expressed as mean (SD) or median (Q1–Q3). * Statistically significant *p*; *p*-values proceeding from multivariate tests (mixed ANOVA) showing the time effect within each group. The pVAS values after exercise protocol for control group were obtained from the assessments at 2′ after the dry needling, as participants did not experience any change. pVAS, visual analog scale for postneedling soreness; DN, dry needling.

**Table 3:** Change in pressure pain threshold (kg/cm^2^) over time within each group.

	Baseline	2 min after	Post Exercise	24 h after	48 h after	Sig.
Eccentric Exercise Group	3.87 (1.34)	3.08 (2.38–4.60)	3.86 (1.78)	2.40 (1.54–4.14)	4.00 (1.83)	*p* < 0.01 *
Concentric Exercise Group	3.11 (2.86–5.05)	2.98 (2.49–4.92)	3.03 (2.37–4.20)	2.83 (1.80–4.81)	3.76 (2.48–6.15)	*p* < 0.01 *
Isometric Exercise Group	5.19 (2.38)	4.49 (1.88)	4.26 (2.99–5.99)	3.82 (1.999)	4.33 (1.91)	*p* < 0.01 *
Control Group	3.87 (1.37)	3.21 (1.39)	3.21 (1.39)	3.64 (1.60)	3.30 (2.40–4.86)	*p* = 0.11

Data were expressed as mean (SD) or median (Q1–Q3). * Statistically significant *p*. *p*-values proceeding from multivariate tests (mixed ANOVA) showing the time effect within each group. The PPT values after exercise protocol for control group were obtained from the assessments at 2′ after the dry needling, as participants did not experience any change. PPT, pressure pain threshold.

## Data Availability

Data are held securely by the research team and may be available upon reasonable request and with relevant approvals in place.
